# Group-Based Online Job Interview Training Program Using Virtual Robot for Individuals With Autism Spectrum Disorders

**DOI:** 10.3389/fpsyt.2021.704564

**Published:** 2022-01-24

**Authors:** Hirokazu Kumazaki, Yuichiro Yoshikawa, Taro Muramatsu, Hideyuki Haraguchi, Hiroko Fujisato, Kazuki Sakai, Yoshio Matsumoto, Hiroshi Ishiguro, Tomiki Sumiyoshi, Masaru Mimura

**Affiliations:** ^1^Department of Preventive Intervention for Psychiatric Disorders, National Center of Neurology and Psychiatry, National Institute of Mental Health, Tokyo, Japan; ^2^Department of Clinical Research on Social Recognition and Memory, Research Center for Child Mental Development, Kanazawa University, Ishikawa, Japan; ^3^Department of Neuropsychiatry, Keio University School of Medicine, Tokyo, Japan; ^4^Department of Systems Innovation, Graduate School of Engineering Science, Osaka University, Osaka, Japan; ^5^Service Robotics Research Group, Intelligent Systems Institute, National Institute of Advanced Industrial Science and Technology, Ibaraki, Japan

**Keywords:** autism spectrum disorders, COVID-19, online job interview, virtual robot, other's perspective

## Abstract

The rapid expansion of online job interviews during the COVID-19 pandemic is expected to continue after the pandemic has subsided. These interviews are a significant barrier for individuals with autism spectrum disorders (ASD). There is little evidence-based training for online job interviews for individuals with ASD, and the development of new trainings is expected. In an effort to facilitate online job interview skill acquisition for individuals with ASD, we developed a group-based online job interview training program using a virtual robot (GOT). In GOT, the interviewer and interviewee are projected as virtual robots on the screen. Five participants were grouped and performed the role of interviewee, interviewer, and evaluator. The participants performed all roles in a random order. Each session consisted of a first job interview session, feedback session, and second job interview session. The participants experienced 25 sessions. Before and after GOT, the participants underwent a mock online job interview with a human professional interviewer (MOH) to evaluate the effect of GOT. In total, 15 individuals with ASD took part in the study. The GOT improved self-confidence, motivation, the understanding of others' perspectives, verbal competence, non-verbal competence, and interview performance scores. There was also a significant increase in the recognition of the importance of the point of view of interviewers and evaluators after the second MOH compared to after the first MOH. Using a VR robot and learning the importance of interview skills by experiencing other perspectives (i.e., viewpoint of interviewer and evaluator) may have sustained their motivation and enabled greater self-confidence. Given the promising results of this study and to draw definitive conclusions regarding the efficacy of virtual reality (VR) robots for mock online job interview training, further studies with larger, more diverse samples of individuals with ASD using a longitudinal design are warranted.

## Introduction

Coronavirus (COVID-19) has slowed things worldwide, but it has not deterred companies from looking for new hires. Online job interviews, though not new, are a useful tool for continued hiring while avoiding the threat of COVID-19. In fact, a host of companies are turning to online job interviews, as they are compelled to cancel in-person meetings amid the spread of the new coronavirus. These interviews also reduce transportation costs, accelerate the interview process, and allow hiring managers to interview non-local candidates. Some employers such as academic institutions would offer applicants the choice of a virtual interview if they were unable to travel after the pandemic has subsided.

Job interviews are significant barriers for individuals with autism spectrum disorders (ASD) (1, 2). These individuals struggle with verbal communication and to convey job-relevant interview content, and they have low self-confidence in their ability to perform in a job interview (3). In addition, they experience difficulties with non-verbal communication, which is directly connected to poor performance during job interviews (2). Online job interviews are not different from in-person job interviewees in the key points required for success. Conveying job-relevant interview content is indispensable. Being confident in front of the screen is also important. Verbal and non-verbal communication still occur during online job interviews. Given these factors, it is natural that individuals with ASD are not good at online or in-person job interviews. As with in-person job interviews, applicants are required to carry out adequate preparation for online job interviews. However, there is little research into the use of online job interviews and evidence-based training for online job interviews for individuals with ASD, and the development of new trainings is expected.

There is concern that individuals with ASD cannot benefit from interventions using real online job interview situations. In our previous experiment, we found that interpersonal tension is high during online interviews for individuals with ASD and that they have low motivation to work in a real online job interview setting. The accumulated intervention literature suggests that social communication training is effective for individuals with high motivation (4–6). Previous studies have demonstrated that many individuals with ASD show motivation and an aptitude for using technology (6–9). These individuals sometimes have a preference for virtual agents (10–12). There is a growing body of research related to the application of virtual worlds, a technology that allows users to practice context-based social and adaptive skills (2, 13–15). In these environments, the user assumes the role of an agent and can safely rehearse initiations and responses. Virtual reality (VR) training offers trainees several advantages, including (1) active participation rather than passive observation, (2) a unique training experience, and (3) low cost and easy access.

A previous study that used a realistic virtual human reported that individuals with ASD looked less frequently at agent peers in an interview setting using VR while talking compared to controls (16). Our preliminary study confirmed that many individuals with ASD are afraid of realistic virtual humans and avoid gazing at these agents because of their realism. When designing objects for use by individuals with ASD, researchers often subscribe to the notion that “simpler is better”; that is, they gravitate toward simple, mechanical objects (8, 17–20). Given these factors, we assume that using a simple virtual agent for training may be adequate for individuals with ASD. Research on virtual exposure with clinical samples indicates that even simple virtual agents can induce a significant increase in anxiety and can be effective for phobic people compared to speaking in front of an empty virtual seminar room (10).

When considering interventions using VR for individuals with ASD, it is important to consider how the eyes of agents are designed because individuals with ASD pay less attention to eyes than individuals with typical development (21). Increasing eye contact is widely acknowledged as an important and promising treatment for individuals with ASD (22, 23). To create useful online job interview training for these individuals, it is important for them to look at the eyes of agents during training. If individuals with ASD can practice eye contact with virtual agents, they may overcome their fear of the gaze of an interviewer and experience decreased anxiety in interview settings.

More importantly, challenges to interview performance are believed to occur because individuals with ASD are impaired in their ability to recognize others' perspectives (24); that is, they are impaired in what has been labeled “theory of mind” (ToM) (25). Therefore, they are unable to understand the effect of their behavior on others (2), which is associated with their low motivation to improve their interview skills (3). In designing an intervention to help individuals with ASD improve their interview skills, it is important not only to teach the interview skills required for such an interaction, but also to improve their understanding of the importance of interview skills. Therefore, interventions from a ToM perspective are needed.

In an effort to facilitate online job interview skill acquisition for individuals with ASD, we developed a group-based online job interview training program using a virtual robot (GOT). In the GOT, five individuals with ASD were assigned to a group. The group consists of one interviewee, one interviewer, and three evaluators. The participants perform all roles in random order. In the GOT, the interviewer and interviewee are projected as virtual robots on the screen. The robots can show a range of simplified expressions that are less complex than those of a real human face. The careful design of the eyes and multiple degrees of freedoms (DoF) dedicated to controlling its field of vision contribute to its rich gaze expressions. By not only performing the role of the interviewee but also the role of the interviewer and evaluator, we expected that the participants could learn others' perspectives (i.e., the perspectives of the interviewer and evaluator). Thus, we consider our system to be effective for online job interviews for individuals with ASD. The present study was carried out to investigate the effectiveness of the GOT.

## Materials and Methods

### Participants

The present study was approved by the ethics committee of Kanazawa University. There was no conflict of interest involved in this study. Participants were recruited by flyers that explained the content of the experiment. After receiving a complete explanation of the study, all participants and their guardians agreed to participate in the study. Written informed consent was obtained from the individuals and/or minors' legal guardian for the publication of any potentially identifiable images or data included in this article. All participants provided written informed consent. The inclusion criteria were as follows: (1) having a diagnosis of ASD based on the Diagnostic and Statistical Manual of Mental Disorders, Fifth Edition (DSM-5) from the supervising study psychiatrist, (2) being male, (3) aged 18–24 years, (4) being unemployed and actively seeking employment, and (5) not taking medication. We excluded female participants because we wanted to avoid fraternizing amongst the participants. Additionally, it is more difficult to gather female participants as ASD is more prevalent in men than in women. At the time of enrollment, the diagnoses of all participants were confirmed by a psychiatrist with more than 15 years of experience in ASD using standardized criteria taken from the Diagnostic Interview for Social and Communication Disorders (DISCO) (26). The DISCO has been reported to have good psychometric properties (27). To exclude other psychiatric diagnoses, the Mini-International Neuropsychiatric Interview (MINI) (28) was administered.

All participants completed the Autism Spectrum Quotient-Japanese version (AQ-J) (29), which is used in the evaluation of ASD-specific behaviors and symptoms. The AQ-J is a short questionnaire with five subscales (social skills, attention switching, attention to detail, imagination, and communication). Previous work with the AQ-J has been replicated across cultures (30) and ages (31). The AQ is sensitive to the broader autism phenotype (32). In this study, we did not set the cut-off according to the AQ-J score and only used DSM-5 and DICSO to diagnose ASD and to judge whether to include participants in our study.

Full-scale IQ scores were obtained by either the Wechsler Intelligence Scale for Children-Fourth Edition or the Wechsler Adult Intelligence Scale-Third Edition.

The severity of social anxiety symptoms was measured using the Liebowitz Social Anxiety Scale (LSAS) (33). This clinician-administered scale consists of 24 items, including 13 items that describe performance situations and 11 items that describe social interaction situations. Each item was separately rated for “fear” and “avoidance” using a four-point categorical scale. According to receiver operating curve analyses, an LSAS score of 30 is correlated with minimal symptoms and is the best cutoff value for distinguishing individuals with and without social anxiety disorder (34).

The ADHD-RS (35) contains 18 items related to inattentive and hyperactive-impulsive symptoms, scored on a four-point scale (0 = never, 1 = sometimes, 2 = often, 3 = very often), and assesses symptom severity over the past week. The total score was computed as the sum of the scores of all 18 items.

### Apparatus

**Figure 1:**
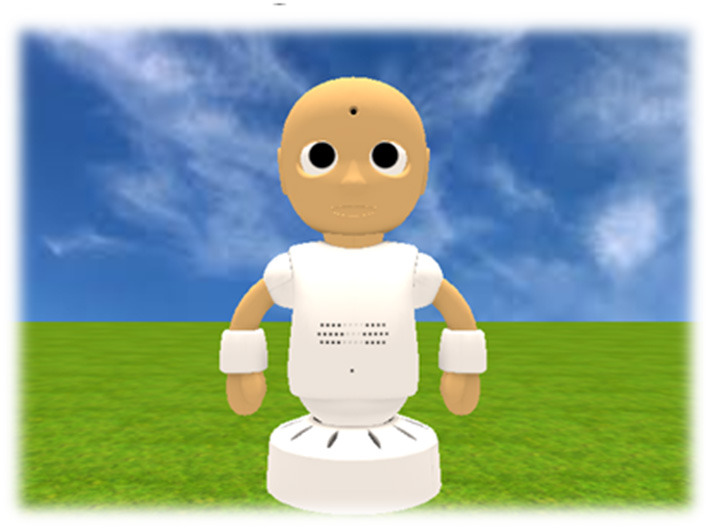
Virtual version of the CommU.

The virtual robot used in this study was a virtual version of a humanoid robot called CommU (36–38) (Figure 1; Vstone Co., Ltd.). CommU has 14 DoFs as follows: waist (2), left shoulder (2), right shoulder (2), neck (3), eyes (3), eyelids (1), and lips (1). The careful design of the eyes and multiple DoFs dedicated to controlling gaze contribute to facial expressions. The face of the CommU can show a range of simplified expressions that are less complex than those of a real human face. Its small and cute appearance is expected to help prevent fearfulness among individuals with ASD. In this experiment, two virtual robots were remotely teleoperated by two participants (i.e., interviewee and interviewer).

On the screen of the interviewee's laptop computer, an avatar operated by the interviewee showed its back while the other avatar operated by the interviewer showed its face so that they faced each other. On the screen of the interviewer's and evaluator's laptop computer, an avatar operated by the interviewer showed its back while the other avatar operated by the interviewee showed its face so that they faced each other. The users' voices were captured by the headsets connected to their laptop computers and transmitted to their interlocutor and evaluators. The captured voices of the users were also used to produce the lip movements of the avatars, and the arm movements of the avatars were designed to resemble human hand gestures during speaking so that the participants could feel as if the voice was produced by the user's avatar.

### Procedures

**Figure 2:**
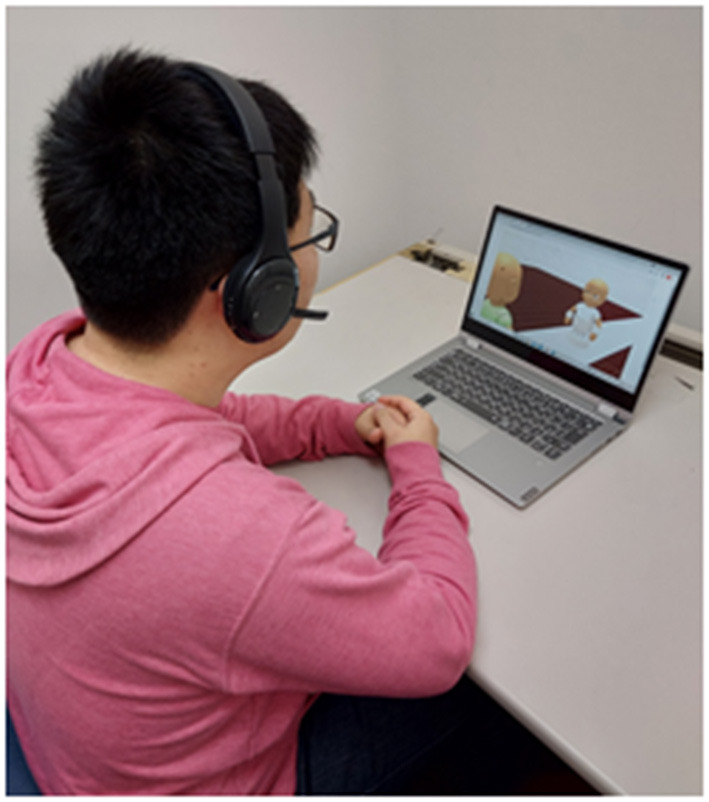
Example of participants participating in mock online job interview.

**Figure 3:**
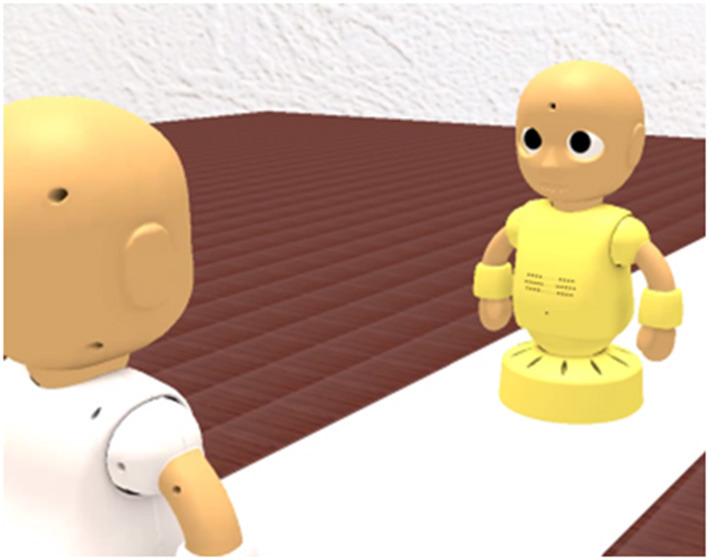
The scene of mock online job interview by virtual robots.

All participants participated the GOT. In the GOT, five participants were grouped and participated in the roles of interviewee, interviewer, and evaluator. Each session consisted of a first job interview session, feedback session, and second job interview session. There were screens in front of each participant (Figure 2). In the first and second job interview sessions, the interviewer and interviewee were projected as virtual robots on the screen and played each role in a mock online job interview (Figure 3). The evaluators are not projected on the screen and observed the mock online job interview on a screen. The participants were placed in individual rooms and were presented similar but different images. The interviewees were shown the image of the interviewer from the viewpoint of the interviewee. The interviewers and evaluators were shown the image of the interviewee from the viewpoint of the interviewer.

In the first job interview session, the interviewees were initially given a document containing recruitment information from which they could select a job, including a data entry clerk, supermarket shelf stocker, custodian, restaurant kitchen assistant, nursing assistant, and paper delivery person. The interviewer asked questions based on prepared lists (see Supplementary Material) and the interviewee answered in a mock online job interview setting.

In the feedback session, no images of virtual robots were presented on the screen. The interviewer and evaluators evaluated the performance of the interviewee based on a prepared scoring sheet that included items on appropriate word use, appropriate question response, speaking calmly, being sincere, being enthusiastic, appropriate eye contact, natural facial expressions, gesturing naturally, appropriate speaking speed, responding with appropriate timing, appropriate vocal volume, and discussion with each other in the online setting. Then, they provided feedback to the interviewee based on their assessment.

Finally, in the second interview session, the interviewee participated in a mock online job interview using virtual robots as in the first setup. The approximate duration of each session was 50 min (i.e., 10 min for the first job interview session, 30 min for the feedback session, 10 min for the second job interview session). The participants completed five sessions in a week to perform every role. The GOT continued for 5 weeks (for a total of 25 sessions).

Before and after the GOT, the participants underwent a mock online job interview with a professional human interviewer (MOH) to evaluate the effect of the GOT.

### Subjective Evaluation

After the first MOH (i.e., MOH before GOT) and the second MOH (i.e., MOH after GOT), we asked the participants to complete a questionnaire about their self-confidence in their interview performance. Items were rated on a seven-point Likert scale (ranging from “1 = not at all comfortable” to 7 = “very comfortable”). We also asked the participants to respond using a five-point Likert scale (1–5) to the question “How motivated are you to learn how to perform in an online job interview?” Responses ranged from 1 (I am not motivated to learn how to perform in online job interviews at all) to 5 (I am very motivated to learn how to perform in online job interviews). In addition, to assess the extent to which each participant in the position of the interviewee understood that their perspective was different from the perspective of the interviewer or evaluator, we asked them to rate, on a scale of 1–5, the extent to which they understood the point of view of the interviewer; 1 meaning, I cannot understand the interviewers' point of view, and 5 being, I can understand the interviewers' point of view perfectly.

### Objective Evaluation

Two trainers independently rated the interview performance of the first and second MOH by watching a video recording of the sessions. The interviewees were scored using a seven-point Likert scale related to verbal competence (i.e., appropriate word use and appropriate question responses), non-verbal competence (i.e., speaking calmly, being sincere, being enthusiastic, appropriate eye contact, natural facial expressions, gesturing naturally, appropriate speaking speed, responding with appropriate timing, and appropriate vocal volume), and interview performance (i.e., sharing things positively, sounding honest, sounding interested in the job, and establishing overall rapport). The ratings ranged from 1 (very poor) to 7 (very excellent). Before the experiment, both raters underwent training (approximately 5 h) on scoring the interviews by watching videos of interview scenes. Both raters were blinded to the filming date (i.e., first MOH or second MOH). The primary rater and secondary rater attained a moderate degree of reliability [intraclass correlation coefficient (ICC) = 0.67] on the interview performance score. The score used in this study was the average score between the primary rater and secondary rater. After the intervention, we asked the participants' supporters, their trainers or job coaches, the following question: “Did the participants learn to understand the point of view of the interviewer after the intervention?”

### Statistical Analysis

We performed the statistical analyses using SPSS version 24.0 (IBM, Armonk, NY, USA). The descriptive statistics of the sample were calculated. The differences in age, IQ, AQ-J, LSAS, and ADHD-RS scores between the groups were analyzed using an independent samples *t*-test. A paired *t*-test was performed to evaluate the improvement of subjective evaluation (i.e., self-confidence, motivation, understanding the point of view of interviewers) and objective evaluation (i.e., appropriate word use, appropriate questions response, speaking calmly, being sincere, being enthusiastic, appropriate eye contact, natural facial expressions, gesturing naturally, appropriate speaking speed, responding with appropriate timing, appropriate vocal volume) between the first and second MOH. Pearson product-moment correlation coefficients were used to explore the relationships between demographic data (i.e., IQ, AQ-J, LSAS, and ADHD-RS) and improvement of subjective evaluation (i.e., self-confidence, motivation, and understanding the point of view of interviewers) and objective evaluation (i.e., appropriate word use, appropriate questions response, speaking calmly, being sincere, being enthusiastic, appropriate eye contact, natural facial expressions, gesturing naturally, appropriate speaking speed, responding with appropriate timing, and appropriate vocal volume). We employed an alpha level of 0.05 for these analyses.

## Results

### Feasibility and Participation

In total, 15 individuals with ASD took part in the study (see Table 1 for participant details). All participants completed the trial procedures without technological challenges or participant distress that would lead to session termination. We carefully observed participant performance and confirmed that all participants were concentrating during the trials and highly motivated from the start to the finish of the experiment.

**Table 1:** Descriptive statistics of participants.

**Characteristics**	***n* = 15**
	***M* (*SD*)**
Age (years)	21.1 (1.8)
Full scale IQ	87.1 (15.7)
AQ-J	32.8 (4.2)
LSAS-J	43.1 (6.8)
ADHD-RS	14.7 (4.5)

*M, mean; SD, standard deviation*.

### Primary Analyses

All participants fully participated in the GOT and seemed to be focused on and engaged in the mock online job interview training using robots.

There was a significant increase in self-confidence after the second MOH compared to after the first MOH (3.27 vs. 2.27) (*p* = 0.002) and in the motivation to learn how to perform online job interviews after the second MOH compared to after the first MOH (3.20 vs. 1.87) (*p* < 0.001). There was also a significant increase in the recognition of the importance of the point of view of interviewers and evaluators after the second MOH compared to after the first MOH (3.47 vs. 1.87) (*p* < 0.001). Details are described in Table 2.

**Table 2:** Means and standard error of the mean of subjective evaluation at first MOH and second MOH.

**Outcome**	**First MOH** ***M* (*SD*)**	**Second MOH** ***M* (*SD*)**	***t***	**Statistics** ***F***	***p***
Self-confidence	2.27 (1.22)	3.27 (0.80)	−3.873	14	0.002^**^
Motivation	1.87 (0.99)	3.20 (0.56)	−4.641	14	<0.001^**^
Understanding the point of interviewer and evaluator	1.87 (0.91)	3.47 (0.64)	−5.527	14	<0.001^**^

*M, mean; SD, standard deviation; MOH, a mock online job interview with human professional interviewer*.

*p < 0.05,

** *p < 0.01*.

There was a significant increase between the first and second MOH in appropriate word use (4.53 vs. 5.37, *p* = 0.006), appropriate question response (4.20 vs. 5.07, *p* = 0.013), speaking calmly (4.20 vs. 4.70, *p* < 0.030), being sincere (4.67 vs. 5.47, *p* < 0.001), being enthusiastic (4.50 vs. 5.37, *p* < 0.001), natural facial expressions (4.07 vs. 4.43, *p* = 0.044), gesturing naturally (4.07 vs. 4.63, *p* = 0.035), appropriate speaking speed (3.70 vs. 4.30, *p* = 0.012), responding with appropriate timing (3.80 vs. 4.60, *p* = 0.001), appropriate vocal volume (4.40 vs. 5.10, *p* = 0.001), sharing things positively (4.70 vs. 5.20, *p* = 0.002), sounding honest (5.10 vs. 5.70, *p* < 0.001), sounding interested in the job (4.23 vs. 5.50, *p* = 0.002), and establishing overall rapport (4.17 vs. 4.87, *p* < 0.001). Details are described in Table 3.

**Table 3:** Means and standard error of the mean of objective evaluation at first MOH and second MOH.

**Outcome**	**First MOH** ***M* (*SD*)**	**Second MOH** ***M* (*SD*)**	***t***	**Statistics** ***F***	***p***
**Verbal Competence**					
Appropriate word use	4.53 (0.85)	5.37 (0.79)	−3.247	14	0.006^**^
Appropriate question response	4.20 (1.08)	5.07 (0.88)	−2.864	14	0.013^*^
**Non-verbal Competence**					
Speaking calmly	4.20 (0.96)	4.70 (1.15)	−2.415	14	0.030^*^
Being sincere	4.67 (0.90)	5.47 (0.74)	−4.773	14	<0.001^^*^^
Being enthusiastic	4.50 (0.93)	5.37 (0.83)	−4.516	14	<0.001^**^
Appropriate eye contact	4.23 (0.96)	4.17 (1.08)	0.323	14	0.751
Natural facial expressions	4.07 (0.59)	4.43 (0.90)	−2.219	14	0.044^*^
Gesturing naturally	4.07 (0.92)	4.63 (1.37)	−2.329	14	0.035^*^
Appropriate speaking speed	3.70 (0.92)	4.30 (1.19)	−2.882	14	0.012^*^
Respond with appropriate timing	3.80 (1.21)	4.60 (1.38)	−4.262	14	0.001^**^
Appropriate vocal volume	4.40 (0.69)	5.10 (0.83)	−4.176	14	0.001^**^
**Interview performance score**					
Sharing things positively	4.70 (0.62)	5.20 (0.65)	−3.873	14	0.002^**^
Sounding honest	5.10 (0.76)	5.70 (0.56)	−4.583	14	<0.001^**^
Sounding interested in the job	4.23 (1.47)	5.50 (1.00)	−3.713	14	0.002^**^
Establishing overall rapport	4.17 (0.84)	4.87 (0.90)	−5.501	14	<0.001^**^

*M, mean; SD, standard deviation; MOH, a mock online job interview with human professional interviewer*.

* *p < 0.05*,

** *p < 0.01*.

In the semi structured interview, the participants' supporters responded to the following prompt: “All students seemed to learn to better understand the point of view of the interviewer.”

There were no significant relationships between demographic data (i.e., IQ, AQ-J, LSAS, and ADHD-RS) and improvement of subjective evaluation (i.e., self-confidence, motivation, understanding the point of view of interviewers) and objective evaluation (i.e., appropriate word use, appropriate questions response, speaking calmly, being sincere, being enthusiastic, appropriate eye contact, natural facial expressions, gesturing naturally, appropriate speaking speed, responding with appropriate timing, and appropriate vocal volume).

## Discussion

In the current study, we assessed the efficacy of the GOT, which is a group-based online job interview training program using virtual robots. The completion rate suggests that participants who received GOT were able to continue to participate in the program without losing motivation. Using a VR robot and learning the importance of interview skills by experiencing other perspectives (i.e., viewpoint of interviewer and evaluator) may have sustained their motivation and enabled greater self-confidence. The GOT helped improve various online job interview skills (i.e., verbal competence, nonverbal competence, and interview performance). Interestingly, this occurred in the absence of specific interview skill training by experts (i.e., improvement based only on practice and feedback by participants with ASD).

In this program, acting as the interviewer using the VR CommU had many advantages compared to conversing face to face in an online setting. Specifically, when conversing face to face, sensory overstimulation from the human interviewer is a serious problem for individuals with ASD, and it interferes with the processing of social signals (39). Furthermore, the technology behind the VR CommU might increase the user's enthusiasm for and concentration on the program. In addition, acting as the interviewee facing the VR CommU may have the advantage of decreasing interpersonal anxiety and promoting intrinsic motivation for the mock online job interview.

In this study, participants performed not only as interviewees but also as interviewers and evaluators, which contributed to their understanding of the perspective of the evaluator. That is, our system may help alleviate the ToM deficit of individuals with ASD in online job interview scenarios. These mechanisms might have enriched the participants' understanding and motivation, led to improvements in their self-confidence and contributed to improvements in various job interview skills.

A previous study suggested that one possible strategy to improve eye contact is not to force individuals with ASD to look others in the eyes but to habituate them to look into the eyes gradually (40). Yoshikawa, Kumazaki (41) reported that interventions using robots and habituating individuals with ASD to make eye contact with robots have the potential to improve eye contact in real interview situations. On the other hand, in this study, the GOT did not improve appropriate eye contact. The study of Yoshikawa, Kumazaki (41) differs from this study in that it used a real robot, which is potentially a more powerful avenue for enhancing skills, whereas this study used a VR robot. In addition, Yoshikawa, Kumazaki (41) used android robot, which are easier to generalize to real-world settings than simple robots (19, 38). In this study, no experts supervised the appropriate use of eye contact, which may also have prevented the GOT from improving appropriate eye contact. Future work using VR android robot, more realistic virtual systems, and expert supervision may further leverage the specific benefits of VR robots.

Several limitations in our study should be acknowledged. First, the number of participants was relatively small. In addition, all participants were male and were of Japanese ethnicity, making the results less generalizable. In Japan, the type of work available differs according to gender. For women, the question “Are you able to carry heavy things?” was unsuitable. To undertake mock online job interview training for women, we would need to change parts of the standard script of the mock online job interview. Future studies involving larger samples that include female participants are needed to provide more meaningful data regarding the potential use of our system. Second, this study is not a controlled study. We did not have a sham training group for comparison with the online virtual robot training. At the time of this experiment, the Japanese government declared a state of emergency due to the spread of COVID-19, so we could not ask participants to participate in control settings. Given that preparing for online job interviews is a pressing issue for individuals with ASD, we had to conduct pilot studies even without a control. In fact, the results of this study provide preliminary evidence supporting the utility of the GOT to some extent. Although there are differences between the first and second interview questions aimed at developing the theme more deeply, it is possible that the improvement between the two interviews resulted from personal growth rather than the intervention. In addition, it is difficult to ascertain whether the improvement is due to the particular characteristics of the virtual program (i.e., specialized eye movements, simple features) or simply from constant rehearsal of interviews and watching participants. Therefore, a multiple baseline design methodology would be more appropriate in this study. The ultimate goal of the program is to enhance communication skills in daily life and to make participants more competitive when searching for employment or volunteer positions. To implement our system, an employment support facility is required. Knowing that the cost of providing care for individuals with ASD is very high (42), supporting these individuals in a competitive position is of great economic importance. To examine whether our program can attain this goal, future studies involving employment support facilities with long-term longitudinal designs are needed. In this study, we could not investigate whether our program translates to actual real-world job interviews. Future studies are needed to determine whether our program can translate into real-world job interviews.

This is the first study to evaluate the effect of mock online job interview training using VR robots. Our program improved self-confidence, motivation, the understanding of others' perspectives, verbal competence, non-verbal competence, and interview performance scores in individuals with ASD. Given the promising results of this study and to draw definitive conclusions regarding the efficacy of VR robots for mock online job interview training, further studies involving individuals with ASD in larger, more diverse samples using a longitudinal design are warranted.

## Data Availability Statement

The raw data supporting the conclusions of this article will be made available by the authors, without undue reservation.

## Ethics Statement

The studies involving human participants were reviewed and approved by the Ethics Committee of Kanazawa University. The patients/participants provided their written informed consent to participate in this study. Written informed consent was obtained from the individuals and/or minors' legal guardian for the publication of any potentially identifiable images or data included in this article.

## Author Contributions

HK designed the study, conducted the experiment, carried out the statistical analyses, analyzed and interpreted the data, and drafted the manuscript. YY, TM, HF, KS, YM, HI, TS, and MM. HH conceived of the study, participated in its design, assisted with data collection and scoring of behavioral measures, analyzed and interpreted the data, and were involved in drafting the manuscript and revised it critically for important intellectual content. MM was involved in approving the final version to be published. All authors read and approved the final manuscript.

## Funding

This work was supported in part by Grants-in-Aid for Scientific Research from the Japan Society for the Promotion of Science (20K20857, 20H00101) and JST, Moonshot R&D Grant Number JPMJMS2011.

## Conflict of Interest

The authors declare that the research was conducted in the absence of any commercial or financial relationships that could be construed as a potential conflict of interest.

## Publisher's Note

All claims expressed in this article are solely those of the authors and do not necessarily represent those of their affiliated organizations, or those of the publisher, the editors and the reviewers. Any product that may be evaluated in this article, or claim that may be made by its manufacturer, is not guaranteed or endorsed by the publisher.
